# Supplementary material for: Incidence of tuberculosis among PLHIV on antiretroviral therapy who initiated isoniazid preventive therapy: A multi-center retrospective cohort study
Source: PLoS One. 2022 May 16;17(5):e0266285. doi: 10.1371/journal.pone.0266285 (PMC9109920; doi:10.1371/journal.pone.0266285)
Supplement: Attachment — Submitted filename: Response to the Reviewers.docx [file pone.0266285.s002.docx]

Dr. Oryokot Boniface

The AIDS Support Organization (TASO) Uganda

P.O. Box 10443 Kampala, Uganda

Email: bonory@gmail.com

To	January 28^th^, 2022

**The Academic Editor,**

*PLoS ONE*

Dear **Editor**,

**RE: Re-Submission of Manuscript; *Incidence of Tuberculosis among PLHIV on Antiretroviral therapy who initiated Isoniazid preventive therapy: a multi-center retrospective cohort study***

On behalf of my team, I am honored to submit a revised version of our original research article, **“*Incidence of Tuberculosis among PLHIV on Antiretroviral therapy who initiated Isoniazid preventive therapy: a multi-center retrospective cohort study”***.

We appreciate the consideration by PLoS One editorial team and the constructive feedback provided for improvement of our manuscript. Our team has made every effort to address and take into consideration all the editor’s and reviewers’ comments. The authors’ responses are detailed in an attached point-by-point rebuttal matrix below and revisions are highlighted as tracked changes in the tracked changes document. We hope that the revised manuscript meets the journal’s publication standards.

|  |  | **COMMENT** | **RESPONSE** |
| --- | --- | --- | --- |
|  |  | **Journal Guidelines** |  |
| 1 | Style requirements | Please ensure that your manuscript meets PLOS ONE's style requirements, including those for file naming. | Thank you: this has been addressed throughout the manuscript |
| 2 | Funding information in text | Please remove any funding-related text from the manuscript and let us know how you would like to update your Funding Statement. Please include your amended statements within your cover letter; we will change the online submission form on your behalf. | Thank you: this has been addressed |
| 3 | Funding statement | Please include your amended statements within your cover letter; we will change the online submission form on your behalf. | Thanks for your concern: this has been addressed |
| 4 | Data availability | If there are no restrictions, please upload the minimal anonymized data set necessary to replicate your study findings as either Supporting Information files or to a stable, public repository and provide us with the relevant URLs, DOIs, or accession numbers. | Thank you: The data has been made available----- |
| 5 | Ethics statement | Your ethics statement should only appear in the Methods section of your manuscript. | Thank you: This was done |
| 6 | Figure captions | Please include a separate caption for each figure in your manuscript. | Thank you. This has been done. |
| 7 | Editor comment | The manuscript could also be shortened somewhat. | Thank you, this has been done |
|  |  | **Reviewer #1** |  |
| 8 | Introduction | Clarification of the national guideline in terms of screening of PLWHIV before initiation of TPT. Was TB ruled out clinically, or with a GeneXpert or with an X-ray? | Thanks for this. TB was ruled out using the WHO 4-symptom screen/ICF Guide, also adapted by our MOH. Eligible PLHIV without symptoms of TB were referred for IPT initiation, while those with TB symptoms were referred for diagnostic testing to exclude active TB before IPT initiation |
| 9 | Methods | The usual cut-off point for multivariate regression is 0.20 or 0.25 and not 0.05 | Thank you. Analysis was repeated considering cut of 0.20 (instead of 0.05) and after model building, the same variables (as previously shown in the multivariate analysis) were retained. No changes in the results |
| 10. | Methods | Specify the type of multivariate analysis: logistic regression or mixed regression Ideally the mixed method is the one recommended to take into account the herogeneity between the centers. But failing that, a logistic regression can be carried out if we consider that the sites are almost identical | Since we estimated the hazard ratio, we used the frailty model which accounts for clustering at centers (heterogeneity between centers) not the logistic regression and not an ordinary mixed model. We have revised the statement to read as “Factors with p-value less than 0.20 at bivariable analysis were all subjected to a multivariable frailty model …” |
| 11 | Results | The age variable: the reference age under 15 years old could be reviewed because in PHIV the child under 15 years old is naturally more vulnerable than an adult subject | Thanks for your suggestion |
| 12 | Abbreviations | There are some unannounced abbreviations. The writing of MOH or MoH? | Thank you. This has been corrected to MOH. |
| 13 | Suggested references | Some suggested references that could be added. | Thank you. These have been considered in the discussion section |
|  |  | **Reviewer #2** |  |
| 14 | General | However, the manuscript would benefit from some rewriting. General suggestions for this follow, but I would advise careful language review. | Thank you. This has been done |
| 15 | Title (and throughout the text): | consider using the term isoniazid preventive therapy (or treatment) as this is what was analyzed, other TPT were not assessed | Thanks. This has been revised accordingly. |
| 16 | Title (and throughout the text): | Also consider using the TBI instead of LTBI, as now suggested by WHO. | Thanks. This has been revised accordingly |
| 17 | Abstract: Introduction | Ugandan setting does not need to be discussed in the background of the abstract, as this is a matter of interest around the globe. | Thank you, this has been revised. |
| 18 | Abstract: Results | Adjusted (instead of crude) HR could be displayed here. | Thank you for your suggestion, and has been adjusted accordingly |
| 19 | Abstract: Conclusion | The conclusion in the abstract is misleading. The authors could highlight here the main messages: incidence rates of TB were low overtime after one course of IPT, and this was mainly due to high completion rates. | Thank you. This has been revised accordingly |
| 20 | Introduction | The introduction could be substantially shortened. They could cite the literature on the specific subject of their analysis | Thank you. This has been done |
| 21 | Introduction and Methods | Additionally, this is a relevant matter outside Uganda as well. Thus, I recommend that the authors move some information on the Uganda national recommendations to the Methods section, under “Setting”. | Thank you, revision has been made accordingly |
| 22 | Introduction | Finally, the Introduction needs updating (WHO report 2020 available). | Thank you for the suggestion and revision has been made accordingly |
| 23 | Methods | please clarify if patients starting or using IPT in the study period were included. Later in the text it seems that this is those starting IPT | We included only PLHIV who initiated IPT from 1^st^/January/2016 to 30^th^/June/2018 |
| 24 | Methods | Please define “stop therapy”. | Thank you. This has been defined |
| 25 | Methods and results | The heading “sample size” is not necessary, the information contained there should be moved to the Results section. | Thank you for your suggestion. This has been revised |
| 26 | Results | Table 1 could be moved to supplement material. | Thank you for the observation. This was done |
| 27 | Methods | The subheading “patient enrolment” could be renamed data extraction. | I agree with the reviewer, rename subheading “patient enrolment” to “Data extraction” |
| 28 | Methods | The exposure variables should be defined in the methods section, after definition of outcomes. | This is noted and has been revised |
| 29 | Methods | It is not clear how the authors “triangulated” data with de-identified registries. | Thank you. This has been revised as below  Where electronic data was missing, patient registers including facility-based TB registers, presumptive TB registers, ART registers, viral load and TPT registers were used. |
| 30 | Methods and Results | Variables cited in the methods section that are not reported in the results section should be suppressed from there (ART status, type of TB, microbiological tests). | Thank you. This has been revised |
| 31 | Methods | Are registered individuals those who were prescribed or those who initiated IPT? Meaning, are those who did not start (primary drop-out) included? | We only included individuals who initiated IPT. |
| 32 | Methods | • Line 47: How can death be based on clinical encounter? | Thank you. Death was based on reports from patient next of kin or caretaker and not clinical encounter |
| 33 | Methods | please use same wording to describe study period | Thank you. This has been adjusted accordingly. |
| 34 | Methods | Was treatment interruption considered at any number of doses taken? Do the authors have information on the mean number of doses taken by those who did not complete IPT? | Thank you. Yes, treatment interruption was considered, regardless of number of doses taken. Information on the mean number of doses taken was not abstracted for this study. |
| 35 | Results | Do the authors have information on cause of death? How was death information collected? I suggest a sensitivity analysis including deaths as a TB outcome (instead of censoring). TB is still the most frequent cause of death among PLH. | Data on cause of death was not available and it was not extracted, instead we collected data on whether one died regardless of the cause (this was all-cause mortality). Actually, death was part of TB outcome (including completed, LTFU, stopped and died) and this was included into the frailty model. But due to small number of deaths (only 4) and yet none of these was diagnosed with TB before dying, the hazard estimate was 2.77e-16 approximated to zero. These results were reported in Table 3. |
| 36 | Results | please inform overall incidence rate (per 100k person-month) as defined in the methods section and shown in the abstract, first paragraph of discussion and table 3. This information is more meaningful than the cumulative incidence. | Thank you for your suggestion. This has been adjusted accordingly. |
| 37 | Results | The p values are unnecessary, as the CI are displayed. | Well, it’s an ongoing argument that when confidence intervals are given, p value shouldn’t be given but other readers still prefer to the see the two results (in fact to some, a p-value is still very important). For this reason, we choose to keep both. |
| 38 | Results | Please consider a “survival curve” figure to illustrate TB cases (I would choose those completing versus those not completing IPT, the difference is impressive, as expected). | Thank you. This was already done, submitted as a supportive document as guided by the journal guidelines |
| 39 | Results | In table 3, do those lost to follow up include all censored? Please add footnote to tables, with abbreviations and this kind of clarification. | Thank you. This was addressed accordingly. |
| 40 | Discussion | The discussion needs to be substantially shortened. The main message seems to be that completed TPT (or preferably, IPT) is associated with sustained protection | Thank you for the suggestion and was acted upon accordingly. |
| 41 | Discussion | Also worthy discussing effect of clinical indicators of severity of HIV disease and absence of effect of other indicators of severity of disease, such as CD4 or type/duration of ART. | Thank you, this was considered. |
| 42 | Discussion | For the sake of clarity for the reader, the authors could calculate the corresponding incidence rates in Tanzania and Ethiopia in the same unit as their findings (cases per 100,000 persons-months). | We appreciate the suggestion and was considered in the discussion section |
| 43 | Discussion | A striking finding that also needs discussion, and explains the low incidence rate overall of TB, is the extremely high treatment completion rates. | Thank you for this observation. Revision was made as such. |
| 44 | Discussion | An additional strength of the study is the active screening for incident TB in follow up visits. | Thank you. This was added as such. |
| 45 | Conclusion | why do the authors conclude that 3 years would be a potential timing for retreatment, if they found 18m as the median time to TB diagnosis? As the authors discuss, they do not have data on re-exposure, and it is not reasonable to speculate, based on their results, a potential timing for retreatment. | Thank you for this observation. It has been accordingly revised. |
| 46 | Conclusion | I also disagree that the authors identified populations who might benefit from retreatment, this was not evaluated | Thank you and the authors have revised this as such. |
| 47 | Conclusion | Conclusions should be restricted to what was evaluated, anything else can be discussed, speculated, but not concluded. | Thank you and paper was revised accordingly. |
| 48 | Abbreviations | Please verify all abbreviations. TASO, cHR, WHO are used in abstract without definition. In the main text, other non-defined abbreviations appear, such as ART and aHR, please check all. | Thank you for the suggestion. Revision has been accordingly made. |
| 49 | General | Consider changing “stopping” to discontinuation of treatment. | Thank you for the suggestion. |
| 50 | Abstract | Also, in the abstract, no space between numbers and the for dates. (1st, not 1 st) | This was addressed. |
| 51 | General | I suggest changing patients enrolled to patients initiated on IPT. | Thank you and this has been revised as well |
| 52 | Results | Line 126 – should be in results (exclusion) | Thank you for the observation |
| 53 | General | Many words with unjustified capital letters, such as the title and the variables in the Methods section | Thank you for the observation. Revision was made. |
| 54 | Discussion | The discussion states “In the present study” at least 5 times. This is not necessary. The reader will know you are discussing your results. | Thank you for the observation. This has been addressed as suggested. |
| 55 |  | L. 318 – discussed highlighted | Thank you. This has been addressed |

We appreciate the corresponding academic editor, Kevin Schwartzman for a job well done, thus far. Finally, we would like to acknowledge USAID/CDC funding of the TASO COE HIV/TB program activities, through the different implementing mechanisms.

Thank you for your consideration!

Dr. Oryokot Bonniface

The AIDS Support Organization (TASO) Uganda
