# Supplementary material for: Incidence of tuberculosis among PLHIV on antiretroviral therapy who initiated isoniazid preventive therapy: A multi-center retrospective cohort study
Source: PLoS One. 2022 May 16;17(5):e0266285. doi: 10.1371/journal.pone.0266285 (PMC9109920; doi:10.1371/journal.pone.0266285)
Supplement: Attachment — Submitted filename: Response to Reviewers.docx [file pone.0266285.s003.docx]

The AIDS Support Organization (TASO) Uganda

P.O. Box 10443 Kampala, Uganda

Email: bonory@gmail.com

To	March 8^th^, 2022

**The Academic Editor,**

*PLoS ONE*

Dear **Editor**,

**RE: Re-Submission of Manuscript; *Incidence of Tuberculosis among PLHIV on Antiretroviral therapy who initiated Isoniazid preventive therapy: a multi-center retrospective cohort study***

On behalf of my team, I am honored to submit a revised version of our original research article, **“*Incidence of Tuberculosis among PLHIV on Antiretroviral therapy who initiated Isoniazid preventive therapy: a multi-center retrospective cohort study”***.

We appreciate the consideration by PLoS One editorial team and the constructive feedback provided for improvement of our manuscript. Our team has made every effort to address and take into consideration all the editor’s and reviewers’ comments. The authors’ responses are detailed in an attached point-by-point rebuttal matrix below and revisions are highlighted as tracked changes in the tracked changes document. We hope that the revised manuscript meets the journal’s publication standards.

| Reviewers’ & Editor’s issues | Author response |
| --- | --- |
| Please review your reference list to ensure that it is complete and correct | All references have been reviewed for completeness and correctness, using Vancouver referencing style  Ref # 6 was incorrect. This was revised and corrected appropriately  Ref #19 was incorrect and has been removed from the list and intext citation.  Ref # 37 was a duplicate of #22. Only one was retained  Ref #21 was improved  Ref# 29 & 30 were incorrect. These have been effectively corrected  All the other references without URLs have been adjusted accordingly. |
| It would be desirable to justify the choice of the age of less than 15 years as a reference in the multivariate analysis method | Thank you for this concern. Our choice was arbitrary, since this was the lowest age category in the series. |
| Incident TB has been attributed to new infections more than reactivation of old infections by many. Your data seem to show the contrary. Anyway, it would be interesting to cite this controversy in your discussion (one sentence) as you have data from the high transmission setting. | Thank you for the suggestion. This has been done. Lines 234-236; 271-273. Ref. Geremew et al |
| Some capital letters still exist | Thank you for raising this. We have revised it appropriately. |
| MoH still appears | Thank you for identifying this one. We have addressed it as well-line 135 |
| That followed by another that | Thank you for this observation. It has been revised accordingly. Line 300 |
| Reviewer 1 had suggested some publications. | Thank you for the concern. However, both publications have already been referenced in the manuscript. #13 & 12. Additional citations included in Lines 68-69 |

We appreciate the corresponding academic editor, Kevin Schwartzman for a job well done, thus far. Finally, we would like to acknowledge USAID/CDC funding of the TASO COE HIV/TB program activities, through the different implementing mechanisms.

Thank you for your consideration!

Dr. Oryokot Bonniface

The AIDS Support Organization (TASO) Uganda
